# Quality of intrapartum care assessed by women with fear of birth who received care in a midwifery continuity model in a rural area of Sweden- a longitudinal cohort study

**DOI:** 10.1186/s12884-026-08914-8

**Published:** 2026-03-09

**Authors:** Ingegerd Hildingsson, Klockar Linda Nääs, Ingela Wiklund, Margareta Johansson

**Affiliations:** 1 Department of Health Science, Mid Sweden University, Holmgatan 10, Sundsvall, 85170 Sweden; 2 Institution of Health & Welfare, Dalarna University, Högskolegatan 2, Falun, 79188 Sweden; 3 Department of Women’s and Children’s Health, Uppsala University, Dag Hammarskjöld’s gata 14 B, Uppsala, 75185 Sweden

**Keywords:** Continuity models, Fear of birth, Intrapartum care, Rural, Quality

## Abstract

**Background:**

The advantages of midwifery continuity models of care during pregnancy, birth, and the postnatal period demonstrated in ‘low-risk’ women may also benefit those with fear of birth. The purpose of the study was to explore women’s assessment of the quality of intrapartum care when participating in a midwifery continuity of care (MCoC) project in a rural area of Sweden. An additional aim was to explore if the care differed between women who had a known midwife during labour and birth, versus not.

**Methods:**

A prospective longitudinal cohort study including 142 women with fear of birth who participated in an MCoC project was performed. Data was collected via two online questionnaires, gathering background data and birth related information. The instrument ‘Quality from the Patients’ Perspective’ was used to assess the perceived reality and the subjective importance of intrapartum care content.

**Results:**

Deficiencies between perceived reality and subjective importance were found in the following aspects of intrapartum care: perception of control (50%), involvement in decision-making (26%), access to preferred pain relief (26%), breastfeeding support (23%), and information about labour progress (22%). The midwife’s presence, involvement of the partner, possibilities to talk through the birth and breastfeeding support was rated better than expected. No major differences were found in the quality of intrapartum care, in relation to continuity.

A total of 75% had a known midwife during labour and birth and were more likely to have had labour induced (aOR 5.31, 95% CI 1.68–16.79), to have used the method ‘Give birth without fear’ (aOR 3.21, 95% CI 1.05–9.83), and to have received an epidural (aOR 3.85, 95% CI 1.51–9.82), compared to women without a known midwife assisting.

**Conclusion:**

The quality of intrapartum care was assessed both deficient and excessive. Few differences were found between women who had a known midwife or not, but women with fear of childbirth who experienced continuity with a known midwife were exposed to more interventions, following Swedish regime to encourage women to have a vaginal birth. Aligning care content with women’s needs and values remains essential to improve satisfaction and outcomes in intrapartum care.

## Background

Pregnant women living in rural or remote areas often lack access to maternity services, due to long distances to health care facilities [1]. Long distance to labour wards has been associated with increased birth interventions and complications [2-4] and fright of ‘giving birth on the road’, [4, 5] and might be one of the reasons behind fear of birth.

Fear of birth has been recognized in Swedish health care regions since the 1990ies [6]. For women with fear of birth, all Swedish hospitals offer counselling where midwives, in collaboration with obstetricians and psychologists, meet with women during 2–3 visits, depending on the woman’s history and needs. It is common to offer women with fear of birth a “birth contract,” a kind of individualized care plan, which may include planning for induction of labour, early pain management, and the possibility to convert to caesarean section if vaginal birth becomes psychologically unmanageable [7]. It is not, however, common that the counselling midwives are those who assist the women they have met during counselling, but if that happens, women are very satisfied with this option and value continuity of care [8], and have reported a better experience of birth and a diminished fear, compared to women who received counselling only [9].

Research on midwifery continuity models have showed fewer medical interventions, and studies consistently report that women value midwifery continuity of care (MCoC) [10–12]. MCoC is a model in which a woman receives most or all of her pregnancy, birth, and postnatal care from the same midwife or a small team of midwives. The core principle is to foster a continuous and trusting relationship between the woman and her midwife, rather than fragmenting care across various providers [10, 11]. A Cochrane review comparing midwife-led continuity models with other care models (shared, medically led, or fragmented) found that MCoC was associated with increased rates of spontaneous vaginal birth and reduced rates of instrumental births (forceps/vacuum). Use of regional analgesia (e.g., epidural) was significantly lower, and there were fewer episiotomies and preterm births among women in MCoC models [11]. In a qualitative systematic review, researchers summarized findings from numerous studies on what childbearing women care about most. Women expressed a desire for birth experiences that are both clinically safe and psychologically secure, grounded in their cultural and personal expectations. They emphasized the importance of receiving practical and emotional support from partners, companions, and especially from competent, reassuring, and kind clinical staff. A sense of control and active participation was also highly valued—even when medical interventions became necessary [13, 14]. While most studies in the Cochrane review [11] focused on women with low risk of complications, there may also be benefits for more vulnerable groups, such as women with fear of childbirth or mental health issues. This is supported by The World Health Organization’s (WHO) call for a global expansion of midwifery models of care [15]. This notion also aligns well with recommendations from the Swedish National Board of Health and Welfare, who recommends focusing on vulnerable populations such as women with fear or birth, when introducing MCoC models [16]. International studies have also examined MCoC in socioeconomically disadvantaged populations, underscoring the need for tailored interventions to meet the specific needs of vulnerable pregnant women [17–19].

### Context of care

Swedish maternity services are divided into primary health care in outpatient clinics, where antenatal care usually commence, with a midwife as the main caregiver. Intrapartum care typically takes place within specialist care in hospitals, and few alternatives to high-technological labour wards are rare. Midwives usually work either in antenatal or intrapartum/postpartum care. In recent years, many labour wards in Sweden have been closed, and birth facilities centralized. This has led to longer hospital stays and increased rates of elective caesarean sections, as shown in a registry study of over 364 000 women [2]. Labour induction due to long travel distances is becoming more common among women in rural areas [3]. Travel times exceeding 60 min to a labour ward have been linked to a higher risk of unplanned out-of-hospital births [4]. Rurality may therefore be a risk factor for fear of childbirth [5]. The few existing MCoC models in rural Sweden have primarily targeted women with fear of childbirth and/or depressive symptoms [1, 5]. These models must also contend with long distances to labour wards and a shortage of midwives available for on-call coverage.

### Problem area

Despite the well-documented benefits of Midwifery Continuity of Care (MCoC) models for pregnant women, there remains a need for more knowledge about vulnerable groups, such as women experiencing fear of birth. Most international MCoC studies focus on urban populations, while information about such models in rural settings is limited.

## Method

### Aim

The purpose of the study was to explore women’s assessment of the quality of intrapartum care when participating in a midwifery continuity of care (MCoC) project in a rural area of Sweden. An additional aim was to explore if the content of care differed between women who had a known midwife assisting during labour and birth, versus not.

### Design

This was a longitudinal cohort study involving women who participated in an MCoC project in a rural area of Sweden.

### Setting

The study was conducted in a Swedish healthcare region with one labour ward, surrounded by a large rural area. The project was initiated by regional policymakers and funded to support four midwives providing care during pregnancy, labour, birth, and the immediate postpartum period. A local project leader was also funded to administer the project. The project was running for two years with the purpose to increasing continuity of care for prioritized women.

### Recruitment of participants

The region’s 29 antenatal clinics, where women book for antenatal care, offer screening for fear of birth and mental health around gestational week 20. Two validated instruments are used in the screening procedure: the Fear of Birth Scale (FOBS) [20, 21] and the Edinburgh Postnatal Depression Scale (EPDS) [22]. Cut-off scores of ≥ 60 on the FOBS [21] and ≥ 13 on the EPDS [23] were used to identify women with fear of birth and depressive symptoms, respectively. Women who met the criteria were informed about the project and, if interested, referred to the MCoC midwives.

### Midwifery continuity of care model

Four midwives were employed to provide antenatal care, parent education, counselling for fear of birth, intrapartum care, and early postpartum support. Before the project started, midwives underwent education sessions about continuity models, treatment for fear of birth and depressive symptoms. These sessions were held by a professor experienced in these areas. Three midwives had their last work experience in intrapartum/postpartum care and were updated on antenatal care, and the midwife with the most recent experience in antenatal care was updated on intrapartum care.

The group of midwives were based in an outpatient clinic located in the same city as the labour ward, where they provided antenatal care. There were around 12 women each month that entered the project. One midwife was primarily responsible for a woman, but all women had the opportunity to meet all midwives in the MCoC team through online sessions or physical meetings. To accommodate women living in rural or remote areas, there was also a possibility to have their physical check-ups during pregnancy at a local health centre, but additional care with the MCoC midwives. The midwives worked on a rotating schedule. Antenatal care with health check-ups, counselling for fear of birth, parent education and birth planning occurred every weekday.

Each weekday one midwife was on-call for birth assistance between 7 am to 5 pm and was replaced by another midwife from 5 pm to 7 am. During the on-call sessions the midwives also made postnatal follow-ups, either in the hospital or online. Weekend on-call shifts covered 24 h. If the midwife worked more than 10 h, she handed over the care to the regular staff on the labour ward.

All women received online counselling, parent education, and birth planning, with the MCoC midwives. A key component was the method *Give Birth Without Fear* [24], which focuses on four principles: Breathing – conscious breathing to manage pain and stress, Relaxation – physical relaxation to support the birth process, Sound – vocalization to release tension and assist with contractions and Power of Thought – positive thinking and mental focus to enhance control and calm. The midwives were scheduled for 24/7 on-call services to assist women during labour and birth. Women were informed about limited availability during summer months due to staffing needs at the labour ward. At the onset of labour, women contacted the on-call midwife, who then provided intrapartum care and arranged follow-up visits.

### Data collection

Data was collected via two online questionnaires distributed through the secure RedCap platform [25]-one during mid-pregnancy and one two months postpartum, between 2022 and 2024. Two reminders were sent to non-responders.

The first questionnaire gathered socio-demographic data including age, cohabitation status, education level, country of birth, number of children, and preferred mode of birth, which served as explanatory variables. The second questionnaire collected birth-related data: onset of labour, mode of birth, pain relief methods, augmentation, and duration of labour.

Quality of intrapartum care was assessed using statements modified from the instrument Quality from the Patients’ Perspective (QPP), to match intrapartum care [26]. These statements addressed aspects such as information about labour progress, support from the midwife and the partner, assistance during the first breastfeeding session, the midwife presence, pain relief, partner involvement, and the opportunity to talk through the birth experience afterwards. Each statement was rated in two dimensions: Perceived Reality (PR): how the care was experienced, and Subjective Importance (SI): how important the aspect was to the respondent. The statements were assessed on 5-point Likert scales ranging from “Totally agree” to “Totally disagree” for PR, and from “Very important” to “Not important” for SI. There was also an option labelled ‘not applicable’ and these responses were omitted from the analysis. Missing data for background variables ranged from 1 to 8, were preferred mode of birth had most missing (*n* = 8). In the comments to this question women wrote tha they couldn’t decide. Missing data was low for the obstetric variables. In the question about labour augmentation only those with spontaneous onset or induction of labour were included and those who answered ‘don’t know’ were omitted.

### Data management

Responses from PR and SI were combined into a seven-level index and thereafter grouped into *Deficient Quality of Care*: (options 1–2), *Balanced Quality of Care*: (options 3–5), and *Excessive Quality of Care*: (options 6–7). According to the QPP index developers, if more than 20% of participants rate an aspect as ‘deficient quality of care’, improvement measures should be considered. If more than 20% is rated ‘excessive’, this should be noted [27]. For further analysis, the index was dichotomized into ‘deficient quality of care’ versus ‘no deficient quality of care’.

### Data analysis

Descriptive statistics (frequencies and percentages) were used to present background characteristics and birth-related data. Comparisons were made between women who had a known midwife during labour and birth, or not, using chi-square analysis. Paired sample t-tests were thereafter used to compare mean scores between PR and SI for each aspect of intrapartum care. Independent sample t-tests were then applied to detect differences in PR and SI between women who had a known midwife assisting during labour and birth versus not. Finally, crude and adjusted odds ratios (OR) with 95% confidence intervals (CI) were calculated between women who were assisted by a known midwife during labour and birth, versus not, of the aspects assessed as ‘deficient quality of care’. The analysis was adjusted for birth-related data. Statistical analyses were performed using SPSS version 29. Ethical review was granted through the Swedish Ethics Review Authority diary number: 2023-00293-01 and the study adhered to the Declaration of Helsinki. The STROBE guidelines for observational studies were followed. Written informed consent was obtained from all individual participants included in the study.

## Results

A total of 200 women were included in the MCoC project. Of these, 175 (87.5%) completed the first questionnaire. The second questionnaire was distributed to 190 women, after excluding those who had moved from the area, experienced late miscarriages, or withdrawn from the project. It was completed by 142 women (74%). No significant differences were found between respondents and non-respondents to the follow-up questionnaire in terms of age, parity, antenatal model of care, assistance from a known midwife, or levels of fear of childbirth and depressive symptoms. Women who completed both questionnaires (*n* = 142) were included in the present study.

### Participant characteristics

Table 1 presents background information. Most participants were aged 32 years or older and lived with a partner. The majority (91%) were born in Sweden, and 74% had a university-level education. 39% were expecting their first baby, while 61% had previous children. 18% expressed a preference for caesarean section. There were no background differences between women who had a known midwife assisting during labour and birth or not.

**Table 1:** Background of the participants

	Women in the project
	*n*=142
	*n *(%)
Age groups	
22-31 years	56 (40.6)
32-44 years	82 (59.4)
missing 4	
Civil status	
Living with a partner	132 (95.6)
Not living with a partner	6 (4.4)
missing 3	
Country of birth	
Sweden	126 (90.6)
Other country	13 (9.4)
missing 3	
Level of education	
High school or lower	36 (25.9)
University education	103 (74.1)
missing 3	
Parity	
Primiparas	54 (39.0)
Multiparas	84 (61.0)
missing 4	
Birth preference	
Vaginal birth	110 (82.1)
Caesarean section	24 (17.9)
missing 8	

### Birth outcomes

Table 2 shows birth related variables. Overall, 37% of women underwent labour induction, and 38% required augmentation with synthetic oxytocin. The majority (77%) had a vaginal birth, while the caesarean section rate was 17.2% (3.6% elective and 13.6% emergency). More than half of the participants used epidural analgesia, and 89% applied the *Give Birth Without Fear* method.

**Table 2:** Intrapartum care

	All Women in the project	Had a known midwife during labour and birth	No known midwife during labour and birth	Differences between women having a known midwife or not
	*n*=142	*n*=107	*n*=35	*p*-value
	*n* (%)	*n* (%)	*n* (%)	
Onset of labour (n=136)				
Spontaneous	86 (63.2)	58 (55.8)	28 (87.5)	
Induction	50 (36.8)	46 (44.2)	4 (12.5)	<0.001
Lenght of labour (mean, SD) (n=134)	10.70 (16.40)	11.41 (12.02)	8.53 (9.08)	0.209
Perceived lenght of labour (n=138)				
Faster than anticipated	71 (51.4)	51 (48.6)	20 (60.6)	
As anticipated	35 (25.4)	29 (27.6)	6 (18.4)	
Longer than anticipated	32 (23.2)	25 (23.8)	7 (21.2)	0.436
Mode of birth (n=142)				
Vaginal	109 (7.)	80 (74.8)	29 (82.8)	
Instrumental vaginal	8 (5.6)	8 (7.5)	0	
Planned caesarean section	6 (4.2)	4 (3.7)	2 (5.7)	
Emergency caesarean section	19 (13.4)	15 (14.0)	4 (11.4)	0.402
Oxytocin for augmentation (n=129)				
Yes	55 (42.6)	44 (44.0)	11 (37.9)	
No/Don't know	74 (57.4)	56 (56.0)	18 (62.1)	0.671
Pain relief and preparation metods¤				
Give birth without fear'	126 (88.7)	99 (92.5)	27 (77.1)	0.026
Nitrous oxide	111 (78.7)	87 (81.3)	24 (68.6)	0.156
Psychoprophylaxis	120 (84.5)	92 (86.0)	28 (80.0)	0.424
Mental training	96 (67.6)	70 (65.4)	26 (74.3)	0.408
Massage	53 (37.3)	43 (40.2)	10 (28.6)	0.235
Epidural	65 (54.2)	66 (61.7)	11 (31.4)	0.003
Any non-pharmacological methods	123 (86.1)	95 (88.8)	28 (80.0)	0.250

¤ Only those who used respective method completed the questions

In all, 75% had a known midwife assisting during labour and birth (the primary midwife or any of the team midwives). The primary midwife was present during labour and birth in 44%, while 59% had another midwife in the team present the whole or part of the time spent in hospital. Sometimes the primary midwife and one of the other team midwives overlapped. It was also common that women were in contact with the midwife on-call during the latent phase (57%) or during travel to the hospital (21%).

We thereafter compared women who had had a known midwife assisting during labour and birth, versus those who did not. Having a known midwife present during labour and birth was associated with increased likelihood of labour induction, and use of certain pain relief methods. After adjusting for relevant background variables, women assisted by a known midwife were more likely to have had labour induced (aOR 5.31, 95% CI 1.68–16.79, *p* = 0.004), used the *Give Birth Without Fear* method (aOR 3.21, 95% CI 1.05– 9.83, *p* = 0.040), and received an epidural (aOR 3.85, 95% CI 1.51–9.82, *p* = 0.050).

### Assessment of intrapartum care quality

Table 3 presents women’s assessments of the quality of intrapartum care. Paired sample t-tests showed statistically significant differences between PR (Perceived Reality) and SI (Subjective Importance) in the areas of decision-making involvement, perception of control, receiving preferred pain relief, presence of the midwife, and the midwife’s involvement of the partner. In most cases, SI was rated higher than PR, except for the presence of the midwife and the involvement of the partner, where PR exceeded SI.

**Table 3:** Perceived Reality and Subjective Importance of aspects of intrapartum care

	Perceived reality (PR)	Subjective importance (SI)	Paired sample t-test
	mean (SD)	mean (SD)	p-value
The midwife I met most of the time gave me the best possible support during labour and birth	3.78 (0.53)	3.87 (0.37)	0.090
I got the best possible support from my partner during labour and birth	3.84 (0.45)	3.84 (0.43)	1.00
I was involved in decision making during labour and birth	3.45 (0.83)	3.62 (0.64)	0.048
I perceived that I had control over my body during labour and birth	2.89 (0.92)	3.55 (0.63)	<0.001
The midwife was present as much as I wanted	3.83 (0.52)	3.63 (0.62)	0.005
I got sufficient information during labour and birth	3.53 (0.78)	3.60 (0.68)	0.324
I received the pain relief method preferred	3.42 (0.89)	3.64 (0.62)	0.014
The midwife made my partner involved during labour and birth	3.76 (0.51)	3.52 (0.74)	<0.001
I had the opportunity to talk through the birth with the assisting midwife	3.34 (0.93)	3.20 (0.93)	0.077
I got the best possible support when I breastfed the first time after birth	2.97 (0.92)	2.75 (1.06)	0.075

Comparisons between women assisted by a known midwife versus those who were not, showed that the former group rated PR significantly higher for “opportunity to talk through the birth afterwards” (mean 3.47 (SD 0.88) vs. 2.58 (SD 1.08), *p* < 0.001) and “midwife’s involvement of the partner” (mean 3.81 (SD 0.45) vs. 3.53 (SD 0.88), *p* = 0.013). For SI, women with a known midwife also rated the importance of discussing the birth afterwards higher (mean 3.33 (SD 0.85) vs. 2.75 (SD 1.07), *p* = 0.003).

### Quality of intrapartum care

Table 4 presents the quality index based on PR and SI. Five aspects of intrapartum care were rated as deficient (> 20%): sense of control (50%), involvement in decision-making (26%), receiving preferred pain relief (26%), breastfeeding support (23%), and information about labour progress (22%). Some aspects were rated as excessive: the midwife’s presence (25%), the midwife’s involvement of the partner (28%), the opportunity to talk through the birth (26%), and breastfeeding support (38%). Only one variable differed between women who had had a known midwife during labour and birth, versus women who did not; the opportunity to talk through the birth afterwards with the assisting midwife, where women who had a known midwife were less likely to perceive that aspect of care as deficient (OR 0.34; 0.12–0.94, *p* = 0.038).

**Table 4:** Women's perception of intrapartum care quality based on an index of Perceived reality and Subjective importance

	Deficient quality*	Balanced quality*	Excessive quality*	Had a known midwife#	Did not have a known	Deficient care when having a known midwife	Deficient care when having a known midwife
	All women in the study				midwife#	midwife	midwife
	*n* (%)	*n* (%)	*n* (%)	%	%	Crude Odds Ratio (95% CI)	Adjusted Odds Ratio (95% CI)¤
The midwife I met most of the time gave me							
the best possible support during labour and birth	17 (12.2)	112 (80.6)	10 (7.2)	13/79/8%	9/88/3%	1.45 (0.39-5.41)	2.32 (0.43-12.30)
I got the best possible support from my partner							
during labour and birth	11 (8.0)	113 (81.9)	14 (10.1)	7/83/10%	15/76/9%	0.33 (0.09-1.19)	0.24 (0.04-1.25)
I was involved in decision making during labour and birth	35 (26.3)	80 (60.2)	18 (13.5)	27/58/15%	23/68/9%	1.29 (0.50-3.34)	2.30 (0.55-9.67)
I perceived that I had control over my body during labour and birth	69 (50.0)	56 (40.6)	13 (9.4)	53/37/10%	39/52/9%	1.75 (0.79-3.90)	2.01 (0.69-5.87)
The midwife was present as much as I wanted	10 (7.6)	89 (67.4)	33 (25.0)	9/68/23%	3/65/32%	3.00 (0.36-27.70)	2.37 (0.20-27.25)
I got sufficient information during labour and birth	29 (22.0)	79 (59.8)	24 (18.2)	23/60/17%	19/58/23%	1.22 (0.45-3.35)	1.50 (0.40-5.61)
I received the pain relief method preferred	31 (26.3)	68 (57.6)	19 (16.1)	27/58/15%	24/56/20%	1.16 (0.41-3.24)	0.94 (0.24-3.61)
The midwife made my partner involved during labour and birth	13 (9.7)	84 (62.7)	37 (27.6)	8/63/29%	16/61/23%	0.43 (0.13-1.45)	0.35 (0.06-1.90)
I had the opportunity to talk through the birth with the assisting midwife	20 (15.6)	75 (58.6)	33 (25.8)	12/61/27%	29/50/21%	0.34 (0.12-0.94)	0.23 (0.05-0.92)
I got the best possible support when I breastfed the first time after birth	26 (22.8)	45 (39.5)	43 (37.7)	25/39/36%	17/41/42%	1.57 (0.53-4.64)	2.50 (0.56-10.98)

*Deficient, balanced and excessive care is based on an index combining perceived reality and subjective importance (QPP, Wilde-Larsson et al, 2001) [27]

#Percentage refers to Deficient/Balanced/Excessive quality of care

¤Adjusted for background characteristics and mode of birth

## Discussion

The main finding of this study was the discrepancy between perceived reality and subjective importance in several aspects of women’s assessment of intrapartum care quality. The result also highlighted that some aspects of intrapartum care quality were viewed as deficient, but also as excessive (better than expected), regardless of whether a known midwife assisted the birth or not. Labour induction and some pain relief methods were more frequently reported if the midwife was known to the woman.

Women’s assessments of intrapartum care revealed discrepancies between perceived reality and subjective importance, particularly regarding information, perception of control, and access to preferred pain relief. These gaps may reflect unmet expectations, which are common in childbirth experiences [28]. External factors, such as rapid labour progression, may limit the ability to meet certain expectations, leading to feelings of reduced control and insufficient information. However, the presence of a known midwife was associated with significantly more positive outcomes in both perceived reality and subjective importance—especially regarding opportunities to talk through the birth afterwards, with the assisting midwife and the midwife’s involvement of the partner during labour and birth. Compared to a previous study of MCoC conducted in a rural area and where the midwives had to travel long distance to two hospitals to assist women [29], and with limited on-call possibilities, the current study showed fewer differences, which might be explained by the stronger integration of the MCoC model with obstetric services in only one hospital where the MCoC-midwives were well known to the rest of the staff. These findings support previous research indicating that relational continuity fosters trust, emotional security, and shared understanding [5, 8]. MCoC with a known midwife has also been shown to reduce birth-related fear and depressive symptoms, highlighting its psychological benefits [30].

The quality index, combining PR and SI, revealed that most women rated their intrapartum mostly balanced. Excessive quality of care reached 25–38% in four variables. This suggests that, overall, the Swedish healthcare system provides a high standard of care with strong levels of patient satisfaction. Still, the quality index revealed that over 20% of women perceived deficiencies in key care aspects, including perception of control, participation in decision-making, pain relief, breastfeeding support, and information. Conversely, some elements—such as midwife presence, birth debriefing, and breastfeeding support—were rated as excessive, suggesting a potential mismatch between institutional care priorities and individual preferences.

The lack of difference between women having had midwifery continuity or not, in the assessed care quality, might be understood as in-built in the study design, since some women gave birth during the summer months where continuity with a known midwife was limited. Nevertheless, all women got support from the MCoC group during pregnancy, in terms of counselling and birth planning. These efforts might have influenced women’s encounters with the regular staff on the labour ward, as their preferences, their treatment for fear of birth and other circumstances were documented in the medical records and the birth plan, giving regular staff the opportunity to fulfil women’s needs. It might also reflect the high standard of intrapartum care overall in Sweden, as previously reported [31].

The elevated rate of inductions among women with a known midwife may reflect the possibility of scheduling labour to ensure midwife presence, thereby reinforcing relational continuity. These findings are in contrast to a large Swedish study of MCoC in the capital region, where the opposite results with reduced induction rate as well as a higher rate of vaginal births was observed among women with FOB who received care within an MCoC model [32].

Alternatively, it may be explained by the Swedish practice of offering women with fear of childbirth an individualized birth plan or “birth contract,” which includes induction of labour, early pain relief, and the option to convert to caesarean section if vaginal birth becomes psychologically unmanageable [7]. Women with fear of childbirth often consider planned caesarean section as a better alternative to vaginal birth [33]. In the present study, 18% preferred caesarean section, which is higher than national studies from Sweden of 7– 8% [34]. Previous research has shown that this preference is more common among women with fear of birth [35]. A randomized controlled trial found that internet-based cognitive therapy or standard counselling reduced caesarean preference during pregnancy, but this preference increased again postpartum when considering future births [35]. These findings suggest that the relatively high induction rate and low caesarean rate in this study may reflect support for vaginal birth while maintaining predictability and psychological safety—consistent with prior research showing that MCoC enhances women’s confidence and sense of security [1, 5, 8]. However, the proportions of caesarean Sect. (17.2%), epidural use (54%), and labour inductions (37%) in this study were comparable to clinic-wide Swedish statistics from 2023 [36].

Women who had a known midwife were also more likely to use the *Give Birth Without Fear* method [24] during labour. Familiarity with techniques practiced during pregnancy appears to enhance involvement and perceived competence during birth. This aligns with previous findings that MCoC improves information sharing, decision-making, and perceived control [8]. This finding, however, warrants a notion, as all women had practiced the method during pregnancy through online sessions with the midwifery group, and the method was integrated in the labour ward’s daily practices, it was therefore surprising that not all women used the method. Also notable was that women assisted by a known midwife were more likely to use epidural analgesia, which contrast the findings from the Cochrane review by Sandall et al. [10, 11], which reported reduced epidural use in MCoC models. This discrepancy may be due to the specific characteristics of the study population—women with fear of childbirth—who are known to have greater needs for effective pain relief [37]. Thus, the higher epidural usage may reflect a more responsive and individualized approach to pain management, rather than overuse. 

Approximately two-thirds of the women in this study lived at a considerable distance from the maternity ward. This is noteworthy, as prior research has linked long travel distances to higher rates of planned caesarean sections and labour inductions [2, 3, 38]. In Sweden, the closure of smaller birthing units over the past 25 years has led to increased concerns about “giving birth on the road” [5, 39]. Having access to a project midwife by telephone during the latent phase of labour or during travel to the hospital was used by several women in the present study. Telephone contact has been highly valued in a previous qualitative study of women living in a rural area and participating in a MCoC model [5] and shows the importance of such options to make women feel secure [40, 41].

### Methodological considerations

This study has several limitations. First, the relatively small sample size limits the generalizability of the findings. Second, the observational design, although longitudinal, lacks a ‘real’ control group, which restricts causal inference. The only comparison group was women who gave birth during the summer, when the midwives were staffing the labour ward which limited the continuity. Nevertheless, 75% had a known midwife, numbers comparable to previous studies without the same restrictions [10, 11].

A randomized controlled trial would have strengthened the study but was not feasible within the two-year project timeframe, especially given that the target population—women with fear of birth or depressive symptoms—represents only about 10% of the birthing population in the region. Additionally, the reliance on self-reported data introduces potential bias. Sweden has very good registers where data is collected from all maternity services. However, there is currently no way to identify women with fear of birth or depressive symptoms that are exposed to MCoC models.

The strength of the study was the longitudinal design, which enabled follow-up with the same participants over time.

## Conclusion

The quality of intrapartum care was assessed as both deficient and excessive, regardless of continuity. Few differences were found between women who had a known midwife or not, but women with fear of childbirth who experienced continuity with a known midwife were exposed to more interventions, following Swedish regime to encourage women to have a vaginal birth. Aligning care content with women’s expressed needs and values remains essential to improve both satisfaction and outcomes in intrapartum care.

## Data Availability

Data is available upon request.

## Abbreviations

MCOC: Midwifery Continuity o Care
FOBS: Fear Of Birth Scale
